# Bariatric Surgery in Moderately Obese Patients: A Prospective Study

**DOI:** 10.1155/2013/276183

**Published:** 2013-12-24

**Authors:** M. Cerci, M. I. Bellini, F. Russo, D. Benavoli, M. Capperucci, A. L. Gaspari, P. Gentileschi

**Affiliations:** Bariatric Surgery Unit, Department of Experimental Medicine and Surgery, University of Rome Tor Vergata, Policlinico di Tor Vergata, Viale Oxford, 81-00133 Roma, Italy

## Abstract

*Introduction*. Moderate obesity (BMI 30–35 kg/m^2^) affects 25% of the western population. The role of bariatric surgery in this context is currently debated, reserved for patients with comorbidity, as an alternative to conservative medical treatment. We describe our experience in moderately obese patients treated with bariatric surgery. *Materials and Methods*. Between September 2011 and September 2012, 25 patients with grade I obesity and comorbidities underwent bariatric surgery: preoperative mean BMI 33.2 kg/m^2^, 10 males, mean age 42 years. In presence of type 2 diabetes mellitus (T2DM) (56%), gastric bypass was performed; in cases with hypertension (64%) and obstructive sleep apnea (OSA) (12%), sleeve gastrectomy was performed. All operations were performed laparoscopically. *Results*. Mean follow-up was 12.4 months. A postoperative complication occurred: bleeding from the trocar site was resolved with surgery in local anesthesia. Reduction in average BMI was 6 points, with a value of 27.2 kg/m^2^. Of the 14 patients with T2DM, 12 (86%) discontinued medical therapy because of a normalization of glycemia. Of the 16 patients with arterial hypertension, 14 (87%) showed remission and 2 (13%) improvement. Complete remission was observed in patients with OSAS. *Conclusions*. The results of our study support the validity of bariatric surgery in patients with BMI 30–35 kg/m^2^. Our opinion is that, in the future, bariatric surgery could be successful in selected cases of moderately obese patients.

## 1. Introduction

Obesity is a world epidemic with remarkable sanitary, social, and economic consequences. Clinically, severe or morbid obesity is defined as values of BMI in the Class III (BMI ≥ 40 kg/m^2^) and Class II (35 ≤ BMI ≥ 39.9 kg/m^2^ in the presence of comorbidities). Obesity is associated with an increased hazard ratio for all-cause mortality [1], as well as significant comorbidity [2].

According to different studies, 25% of the western population is affected by some degree of obesity that can be defined as moderate or Class I obesity (BMI between 30–35 kg/m^2^). According to the literature, also patients with Class I obesity have a definite risk of significant comorbidity, such as diabetes mellitus, hypertension, dyslipidemia, obstructive sleep apnea syndrome, and mortality [3]. Other studies suggest that the clinical picture of patients affected by Class I obesity can be improved as well as in patients with severe obesity by bariatric surgery, with weight loss and resolution of comorbidities.

The present prospective study aimed to investigate the improvements or remission of type II diabetes, hypertension, and obstructive sleep apnea in a series of Class I obese patients submitted to a bariatric surgical procedure at our institution [4].

## 2. Material and Methods

From September 2011 to September 2012, 25 patients affected by Class I obesity with associated comorbidities were enrolled in the study and submitted to bariatric surgery. The study group consisted of 15 women and 10 men with mean age of 42.2 years (range, from 28 to 59 years) and a mean BMI of 33.2 kg/m^2^ (range, from 30 to 35 kg/m^2^). Of the 25 patients, 14 (56%) had type II diabetes, 16 (64%) had hypertension, 3 (12%) had obstructive sleep apnea syndrome (OSAS), with 8 patients with multiple comorbidities.

All patients were studied preoperatively with a multidisciplinary workup including selected counselling (surgery, psychiatry, nutrition, and anesthesiology), gastrointestinal endoscopy, and complete performance status evaluation. All patients were well informed about the surgical procedure offered (potential advantages, cost-benefit ratio, and postoperative complications or side effects). Roux-en-Y Gastric Bypass (RYGB) was preferentially performed in 14 (56%) patients with T2DM. In the remaining patients, we decided to perform sleeve gastrectomy (SG). All the procedures were performed laparoscopically. In case of SG, a 36 French orogastric tube was routinely used with reinforcement of the shears with matrix of thrombin and fibrin glue; in the other surgical procedures (RYGB), the usual surgical technique was performed, with the creation of a small 15–30 cm^3^ proximal gastric pouch. The jejunum was transected at 50 cm distal to the ligament of Treitz to create the biliopancreatic limb. The distal jejunum was anastomosed to the gastric pouch, and the biliopancreatic limb was anastomosed to the alimentary limb 100 cm below the gastrojejunostomy.

A swallow test with gastrografin was performed on the first postoperative day and a liquid diet was immediately started. Patients were discharged as soon as they could walk, drink, and be free of significant clinical complications. Patients were given a liquid diet for two weeks and followed up in our outpatient clinic for one year.

## 3. Results

Mean postoperative follow-up was 12.4 months (range from 7 to 19 months). We had a single episode of postoperative hemorrhage at the trocar site that was treated by surgical ligation of the arterial vessel of the abdominal wall. Mean BMI reduction after surgery at 12-month distance was 6 kg/m^2^ with an average value of 27.2 kg/m^2^ (range from 24.6 to 28.8 kg/m^2^). Of the 14 diabetic patients, 12 (86%) had complete remission (discontinuation of drug therapy and normalization of glycemic blood values). An improvement was seen in 2 of the 16 hypertensive patients, with a complete resolution in all the others. Obstructive sleep apnea was resolved in all 3 patients. The results are shown in Table 1.

## 4. Discussion

Traditional bariatric surgery guidelines, approved in 1991 by the National Institute of Health in USA [5], did not include moderate obesity or Class I obesity (BMI from 30.0 to 34.9 kg/m^2^) as an indication to surgery, excluding a large amount of obese patients (approximately 25%) from an effective therapeutic approach. Unfortunately, in this group of moderately obese patients (MOB) the risk of associated metabolic and cardiovascular disease is consistent [6]: even mortality is high and correspondent life expectancy is reduced.

Updated guidelines were developed in 2013 by the American Association Of Clinical Endocrinologists, the Obesity Society, and the American Society for Metabolic and Bariatric Surgery [2]. In this extensive evidence-based review, 74 recommendations about bariatric surgery were provided. Main topics included the role of sleeve gastrectomy, bariatric surgery in patients with Type II Diabetes, copper deficiency, informed consent, behavioral issues, and bariatric surgery for patients with mild obesity. As for the latter, there is emerging data supporting the following concept: patients with BMI of 30–34.9 kg/m^2^ with diabetes or metabolic syndrome may also be offered a bariatric procedure, although current evidence is limited by the number of subjects studied and lack of long-term data demonstrating net benefit. There is insufficient evidence for recommending a bariatric surgical procedure specifically for glycemic control alone, lipid lowering alone, or cardiovascular disease risk reduction alone, independent of BMI criteria [1, 7].

A recent systematic review [4] assessed the association between bariatric surgery versus nonsurgical treatment and weight loss and glycemic control among patients with diabetes and BMI of 30 to 35 kg/m^2^. The authors conclude that bariatric surgery in this setting produces greater short-term weight loss and better intermediate glucose outcomes. Even in this paper the authors state that the evidence about the appropriate use of bariatric surgery in this population is insufficient and more data are needed.

For this, reason, we believe that our series is important because it is based on a prospective study in moderately obese patients with different comorbidities (not only diabetes) submitted to bariatric surgery. This approach, in our study, was feasible and safe.

Short-term efficacy of Laparoscopic Adjustable Gastric Banding (LAGB) in mild to moderate obesity and type 2 diabetes with Class I obesity has been demonstrated in clinical studies [6, 8–10], leading the Food and Drug Administration to approve the use of this procedure for patients with a BMI of 30 to 35 kg/m^2^ with type 2 diabetes or other obesity-related comorbidities [11]. The purpose of our study was then focused on the use of more effective procedures (SG and RYGB) in this patient population, following some pioneer study already described in the literature.

In particular, reported studies suggest that surgical bariatric procedures (LAGB, RYGB, and/or SG) might be useful in moderately obese patients with severe comorbidities, in terms of weight loss and resolution of cardiovascular or metabolic comorbidities, with particular attention to type 2 diabetes mellitus (T2DM).

A recent meta-analysis [12] reviews 16 different retrospective clinical investigations (343 patients) focused on moderate obesity and T2DM. Patients were offered different types of bariatric surgery procedures. The meta-analysis concluded that bariatric surgical procedures performed in these patients definitely improved or normalized glycemic control in T2DM patients after 6 months, prior to weight loss with a very low postoperative morbidity rate (0.29%). Interestingly, according to this meta-analysis, these patients showed a good postoperative glycemic control (fasting and postprandial) immediately after surgery: the authors of this study concluded that this shocking result was obtained not because of weight loss but only because of the drastic change in the anatomy of the upper digestive tract determined by the bariatric surgical procedure, mainly related to the modified physiopathology of the intestinal hormonal and neurotransmitter homeostasis.

In 2010, Diabetic Surgery Summit (DSS) approved bariatric surgery as an alternative to achieve optimal and quick glycemic control in moderately obese patients, whereas other conservative medical options showed to be ineffective [13]. Historically, medical literature showed dramatic improvement in fasting and postprandial glycemic control of nonobese patients that underwent different upper gastrointestinal surgeries similar to modern bariatric surgery procedures [12]. In this situations, different surgical procedures addressed to the reconstruction of the digestive GI tract usually lead to different levels of optimal glycemic control in diabetic patients (fasting and postprandial). For example, RYGB procedures are generally associated with a better glycemic control when compared to gastric restrictive surgeries like LAGB and/or SG [12].

LAGB is a restrictive minimally invasive bariatric procedure: its operative mortality is considerably low (0.05%) and this was the principal reason why LAGB was the first procedure offered to normoglycemic MOB patients: weight reduction obtained by LAGB was very good, not significantly different from the results obtained in type II and type III morbidly obese patients (BMI > 35–40 kg/m^2^).

LAGB, according to Dixon et al. [14] in a randomized trial, was associated with a definite better glycemic control compared to conservative medical therapy: remission of type 2 diabetes was achieved by 73% of patients in the surgical group and 13% in the conventional-therapy group, after 2-year follow-up. Abbatini et al. [15] reported SG compared to medical treatment in MOB diabetic patients and observed a complete remission of T2DM in 88.8% of patients submitted to SG versus 0% in medical therapy group. Cohen et al. [16] reported a similar rate (90%) of glycemic normalization in MOB patients that underwent RYGB.

In a recent study, Choi et al. [17] investigated the use of LAGB in 66 Class I obese patients compared to 438 severe obese patients: the mean percentage of excess weight loss was 20.3% ± 9.0%, 28.5% ± 14.0%, 44.7% ± 19.3%, and 42.2% ± 33.7% at 3, 6, 12, and 18 months, respectively. This was not significantly different from the excess weight loss in the control group, except for at 12 months. Both groups showed similar improvement of most comorbidities. Both groups of patients showed similar postoperative improvement in associated cardiovascular and metabolic comorbidities: overall postoperative morbidity rate was 6% in Class I obese patients (4 patients experienced slippages of the banding (2), banding erosion (1), and port seroma (1)). No mortality was observed in both groups.

Previously, Parikh et al. [6], between 1998 and 2004, enrolled 93 moderately obese patients (mean BMI of 32.7 kg/m^2^) and submitted them to LAGB. Of the 93 patients (76 women and 17 men), 42 (45%) had comorbidities (T2DM, HBP, OSAS, and Chronic Obstructive Pulmonary Disease (COPD)). At three years distance, postoperative BMI reached a mean value of 27.6 kg/m^2^ and most of the associated preoperative comorbidities disappeared or showed a considerable remission/improvement, 88% approximately. Interestingly, in this study no postoperative mortality was observed.

All these studies point out that normalization of glycemic control in MOB patients is probably independent from weight loss, being more often correlated to the surgically induced modification of secretion of upper digestive GI regulatory hormones [18].

Laparoscopic Sleeve Gastrectomy (SG) is usually offered to severely obese patients in different clinical trials and it showed considerable clinical advantages if compared to LAGB. In MOB patients, on the other hand, SG is rarely offered in the literature. Excellent preliminary results on the first 23 patients, in a trial of 79 patients submitted to of SG beginning from April 2007, with a mean follow-up of 6 months, have been reported by a Swedish study [19]. The percentage of excess weight loss, after only 6 months from the intervention, has been of 100%, with a reduction of the BMI, from an initial mean value of 33.8 kg/m^2^ (range 30.8–35.0 kg/m^2^), to 25 kg/m^2^ (range 20–29). Furthermore, associated metabolic and cardiovascular comorbidities showed a considerable remission or resolution. Unfortunately, in this study two major postoperative bleeding episodes and anastomotic leak in the upper portion of the gastric tube requiring a stenting procedure were postoperatively observed. Minor postoperative complications, usually self limiting, were persistent incisional scar pain (1) and minor abdominal bleeding not requiring reoperative surgical procedure (1). No postoperative thromboembolism and mortality were observed.

Then, bariatric surgery in MOB might be associated with significant advantages in terms of cost-effectiveness especially during a long term period of observational follow-up.

A recent investigation, [20] conducted by Southampton Health Technology Assessments Centre SHTAC, has evaluated cost-effectiveness of bariatric surgery (laparoscopic gastric banding) in patients affected by moderate obesity, comparing these results to the conservative dietologic medical approach. This study involved two different clinical trials and it clearly demonstrated a superiority of laparoscopic gastric banding in terms of weight reduction in comparison with conservative medical therapy. In this study, surgery, even if apparently more expensive in the short term than conservative medical therapy, was definitely more promising and effective in the medium and long-term period mainly because of the cost reduction of medical therapy of comorbidities.

All patients in our study experienced a safe postoperative period and a good weight loss over a 1-year follow-up. Most of them are now free from medical therapy and still losing a certain amount of weight.

## 5. Conclusions

The results of our study, although initial and limited by the small number of patients, seem to confirm the feasibility and safety of bariatric surgical procedures in MOB with a BMI of 30–35 kg/m^2^. A larger number of patients are needed in the future as well as clinical prospective studies with well-designed end points in terms of associated metabolic and cardiovascular comorbidities and life expectancy.

Important questions that still need to be answered regard failure of normalization of the glycemic control (fasting and postprandial) in a residual but persistent subset of patients and optimal timing of the surgical bariatric procedure offered to MOB patients.

In our opinion, bariatric surgery, either restrictive (laparoscopic SG) or malabsorptive (RYGB), might be successfully proposed in moderate or Class I properly selected patients, considering the high failure rate of the conservative medical therapy in these settings.

## Figures and Tables

**Table 1:** BMI and comorbidities after bariatric surgery.

	At baseline	Postoperative
Mean BMI	33.2 kg/m^2^	27.2 kg/m^2^
Diabetes	14 pz (56%)	2 pz (4%)
Hypertension	16 pz (64%)	2 pz (4%)
OSAS	3 pz (12%)	0 (100%)
